# The evolution of basal progenitors in the developing non-mammalian brain

**DOI:** 10.1242/dev.127100

**Published:** 2016-01-01

**Authors:** Tadashi Nomura, Chiaki Ohtaka-Maruyama, Wataru Yamashita, Yoshio Wakamatsu, Yasunori Murakami, Federico Calegari, Kunihiro Suzuki, Hitoshi Gotoh, Katsuhiko Ono

**Affiliations:** 1 Developmental Neurobiology, Kyoto Prefectural University of Medicine, INAMORI Memorial Building, 1-5 Shimogamo-hangi cho, Sakyo-ku, Kyoto 606-0823, Japan; 2 Japan Science and Technology Agency (JST), PRESTO, 4-1-8 Honcho, Kawaguchi, Saitama 332-0012, Japan; 3 Neural Network Project, Department of Neural Development and Regeneration, Tokyo Metropolitan Institute of Medical Science, 2-1-6, Kamikitazawa, Setagaya-ku, Tokyo 156-8506, Japan; 4 Department of Developmental Neuroscience, United Centers for Advanced Research and Translational Medicine (ART), Tohoku University Graduate School of Medicine, Sendai, Miyagi 980-8575, Japan; 5 Graduate School of Science and Engineering, Ehime University, Matsuyama, Ehime 790-8577, Japan; 6 DFG-Centre for Regenerative Therapies Dresden, Faculty of Medicine, TUD, Fetscherstrasse 105, 01307 Dresden, Germany; 7 Department of Biology, Nihon University School of Dentistry at Matsudo, Chiba 271-8587, Japan; 8 Division of Companion Diagnostics, Department of Pathology and Microbiology, Nihon University School of Medicine, Tokyo 173-8610, Japan

**Keywords:** Tbr2, Eomes, Gpsm2, Amniote, Basal progenitor, Evolution, Pallium, Chicken, Mouse, Opossum, Turtle, Axolotl, *Xenopus*

## Abstract

The amplification of distinct neural stem/progenitor cell subtypes during embryogenesis is essential for the intricate brain structures present in various vertebrate species. For example, in both mammals and birds, proliferative neuronal progenitors transiently appear on the basal side of the ventricular zone of the telencephalon (basal progenitors), where they contribute to the enlargement of the neocortex and its homologous structures. In placental mammals, this proliferative cell population can be subdivided into several groups that include Tbr2^+^ intermediate progenitors and basal radial glial cells (bRGs). Here, we report that basal progenitors in the developing avian pallium show unique morphological and molecular characteristics that resemble the characteristics of bRGs, a progenitor population that is abundant in gyrencephalic mammalian neocortex. Manipulation of LGN (Leu-Gly-Asn repeat-enriched protein) and Cdk4/cyclin D1, both essential regulators of neural progenitor dynamics, revealed that basal progenitors and Tbr2^+^ cells are distinct cell lineages in the developing avian telencephalon. Furthermore, we identified a small population of subapical mitotic cells in the developing brains of a wide variety of amniotes and amphibians. Our results suggest that unique progenitor subtypes are amplified in mammalian and avian lineages by modifying common mechanisms of neural stem/progenitor regulation during amniote brain evolution.

## Abstract (precis)

**Highlighted article:** In the developing chick pallium, a basal progenitor population resembles mammalian cortical basal radial glia, suggesting a more ancient evolutionary origin for this cell type.

## INTRODUCTION

Increased heterogeneity in neural progenitor pools and the precise regulation of progenitor dynamics are essential for the evolution of intricate brain architectures. In the developing mammalian neocortex, two types of neuronal progenitors are distinguished according to their position during mitosis: apical progenitors (APs) divide at the apical surface of the ventricular zone (VZ), whereas basal progenitors (BPs) undergo mitosis in the subventricular zone (SVZ) (Götz and Huttner, 2005; Kriegstein et al., 2006). BPs are also classified into several subtypes based on their morphology, gene expression and fate (Betizeau et al., 2013; Pilz et al., 2013). In the developing mouse neocortex, the majority of BPs are Tbr2^+^ (Tbr2 is also known as Eomes), multipolar intermediate progenitors (Englund et al., 2005; Arnold et al., 2008; Sessa et al., 2008; Vasistha et al., 2014). However, recent studies have described basal radial glial cells (bRGs), a unique BP subtype that maintains radial glial features within the developing mammalian neocortex (Fietz et al., 2010; Hansen et al., 2010; Reillo et al., 2011; Betizeau et al., 2013; Borrell and Götz, 2014). bRGs have been recognized in a variety of mammals, including species with lissencephalic neocortex (Shitamukai et al., 2011; Wang et al., 2011; Garcia-Moreno et al., 2012; Martínez-Cerdeño et al., 2012; Reillo and Borrell, 2012; Dehay et al., 2015), although the evolutionary origin of distinct BP subtypes remains elusive. Here, we report the unique characteristics of basal mitotic cells in the developing avian pallium, and show that these cells resemble the bRGs in the mammalian neocortex. Genetic manipulation revealed that BPs and Tbr2^+^ cells are distinct cell lineages in the developing avian telencephalon. Furthermore, a small population of basal/subapical mitotic cells was detected in the developing brains of a wide variety of tetrapods. These results suggest that BPs are amplified in mammalian and avian lineages by modifying common mechanisms of neural stem/progenitor regulation during amniote brain evolution.

## RESULTS

### BPs and Tbr2^+^ cells are distinct populations in the developing chicken pallium

**Fig. 1.**
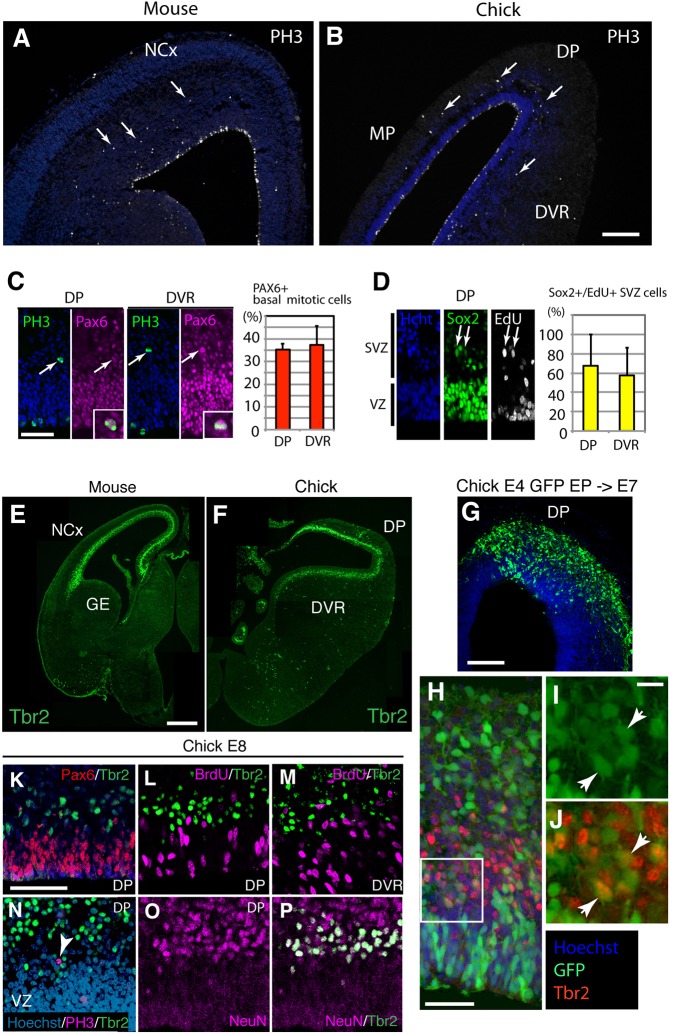
**Distinct characteristics of basal mitotic cells and Tbr2**^+^ **cells in the developing chicken pallium.** (A,B) Distribution of phosphorylated histone H3 (PH3)^+^ cells in the developing mouse (A, E15.5) and chick (B, E8) telencephalon. Arrows indicate mitotic cells on the basal side of the pallia. (C,D) The proportion of the PH3^+^ cells that were also Pax6^+^, Sox2^+^ or EdU^+^ on the basal side of the chicken pallium. Arrows, indicate PH3^+^/Pax6^+^ cells (C, magnified by insets) and Sox2^+^/EdU^+^ cells (D). Mean±s.d. (E,F) Distribution of Tbr2^+^ cells in the developing mouse (E, E12.5) and chick (F, E8) telencephalon. (G-J) Labeling of Tbr2^+^ cells by electroporation (EP) with a GFP expression vector. Electroporation was performed at E4 and the chick embryos were collected at E7. Arrowheads (I,J), GFP^+^/Tbr2^+^ cells. (K-P) Characterization of Tbr2^+^ cells in the developing chick pallium. The sections were stained with anti-Pax6 (K), anti-BrdU (L,M), anti-PH3 (N) or anti-NeuN (O,P) antibodies and an anti-Tbr2 antibody. Arrowhead (N), a PH3^+^ cell that does not overlap with Tbr2. DP, dorsal pallium; DVR, dorsal ventricular ridge; MP, medial pallium; NCx, neocortex; GE, ganglionic eminence; SVZ, subventricular zone; VZ, ventricular zone. Scale bars: 200 µm in B (for A,B), E (for E,F), G; 50 µm in C (for C,D), K (for K-P); 10 µm in I (for I,J).

Previous studies have shown that basal mitotic cells are also present in the developing avian pallium (Cheung et al., 2007; Abdel-Mannan et al., 2008; Charvet et al., 2009; Striedter and Charvet, 2009), which contains a region that is thought to be homologous to the mammalian neocortex (Puelles, 2001; Striedter, 2005) (Fig. 1A,B). We show that a certain population of chicken basal mitotic cells express neural stem/progenitor cell markers. Phosphorylated histone H3 (PH3)^+^ and 5-ethynyl-2′-deoxyuridine (EdU)^+^ cells were localized in the basal position and were also found to be positive for Pax6 or Sox2 (Fig. 1C,D), which are transcription factors characteristic of neural stem/progenitor cells among various vertebrate species.

Based on the quantification of PH3^+^ cells in the developing E8 chicken pallium, we estimated the proportions of APs and BPs as 64.83±1.95% and 35.16±5.08%, respectively. We also identified that, in the SVZ and mantle zone (MZ) of E8 chicken pallium, 62.49% of EdU^+^ cells were Sox2^+^ and 37.51% were Sox2^−^ [the mean of dorsal pallium (DP) and dorsal ventricular ridge (DVR)], suggesting that the former comprised ‘neural' progenitors, whereas the latter were non-neuronal in lineage (Fig. 1D) (although it is also possible that we underestimated the proportion of basal neural progenitors, because there might be Sox2^−^ neural progenitors in the SVZ and MZ). These data suggest that, among total cycling cells, at least 66% are APs, 21.87% are putative basal neural progenitors and 13.12% are non-neural lineage progenitors (such as endothelial cells). Notably, 24 h after pulse labeling, the majority of EdU^+^ cells were also positive for Tbr1, a marker of differentiated pallial neurons (Fig. S1A,B). The proportion of Tbr1^−^ cells in the SVZ and MZ was 9.47% (the mean of DP and DVR; Fig. S1B), which was consistent with the proportion of Sox2^−^ progenitors (13.12%; *P*=0.27, Chi-square test), suggesting that these cell populations were derived from non-neuronal lineage. In other words, our data implicated that the majority, if not all, basal mitotic cells that expressed Sox2 contributed to the Tbr1^+^ neuronal population in the SVZ and MZ, population in the SVZ and MZ.

Tbr2, a marker for BPs in the developing mammalian cortex, as shown in the embryonic mouse cortex (Fig. 1E,F), is also expressed in the SVZ of the developing chick pallium (Bulfone et al., 1999; Suzuki et al., 2012; Martínez-Cerdeño et al., 2015). *In ovo* electroporation revealed that GFP-labeled cells derived from chicken pallial APs express Tbr2 during the course of their neuronal differentiation (Fig. 1G-J). However, in contrast to what was observed in the mouse neocortex, we rarely detected BrdU-labeled or EdU-labeled cells or PH3^+^ mitotic cells among chicken Tbr2^+^ cells (EdU^+^/Tbr2^+^ cells, 0.49±0.07% at E8 and 0.25±0.35% at E10; PH3^+^/Tbr2^+^ cells, 0.05±0.08% at E8 and 0.15±0.13% at E10; Fig. 1K-N, Fig. S1). Instead, the majority of chicken Tbr2^+^ cells were labeled with an anti-NeuN (Rbfox3) antibody (94.4% of Tbr2^+^ cells were NeuN^+^; Fig. 1O,P). We also confirmed that Tbr2 was rarely expressed in cycling cells in the basal side of the chicken pallium (Tbr2^+^/basal PH3^+^ cells, 0.66±1.15% at E8 and 0.81±0.86% at E10; Tbr2^+^/EdU cells, 3.55±1.41% at E8 and 0.19±0.27% at E10). Thus, Tbr2^+^ cells do not display characteristics of cycling progenitors, which are a distinct population from BPs in the developing chicken pallium.

### Avian BPs retain apical and basal radial fibers during mitosis

**Fig. 2.**
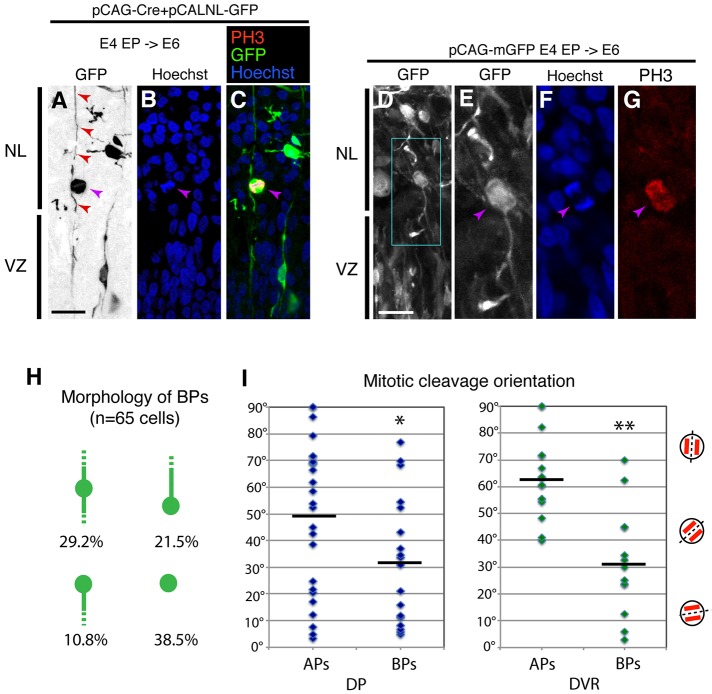
**Radial glial morphology of chicken basal mitotic cells.** (A-G) Visualization of basal mitotic cells in the developing chicken pallium with the pCALNL-GFP vector (A-C) or a membrane-bound GFP vector (D-G). Electroporation was performed at E4 and samples were collected at E6. Note that PH3^+^ basal mitotic cells (purple arrow) extended radial fibers toward both the basal and apical sides of the brain (red arrowheads in A). (D-G) Oblique cleavage plane of basal mitotic cells. (H) Classification of basal mitotic cells based on the presence of apical and/or basal radial fibers. (I) Quantification of the cleavage plane angle relative to the ventricular surface in APs and BPs. Bars indicate mean. *P<0.05, **P<0.01; Student's *t*-test. NL, neuronal layer; VZ, ventricular zone. Scale bars: 10 µm in A (for A-C), D (for D-G).

To analyze the morphology of chicken BPs, we electroporated an expression vector that drives EGFP upon Cre-mediated recombination (pCALNL-GFP). Co-electroporation with the Cre expression vector at a lower concentration reduced the recombination frequency, which allowed us to examine the morphological characteristics of each progenitor (Fig. 2A-C). We also used an expression vector encoding a membrane-bound form of GFP (mGFP) to visualize the cellular processes extending from the BPs (Fig. 2D-G). Interestingly, chicken BPs retained the radial fibers that extended toward the apical and/or basal side of the pallium (Fig. 2A-C,H). Because chicken radial fibers extend along highly curved trajectories (Nomura et al., 2008), we were not able to trace the full extent of the projection of each fiber toward the ventricular or pial surface.

We also determined the cleavage orientation of PH3^+^ mitotic cells and compared the orientations of APs and BPs. In the E8 chicken DP, APs exhibited random cleavage orientation at anaphase, including horizontal, oblique and perpendicular orientations with respect to the ventricular surface (the average angle was 49.1°; Fig. 2I). By contrast, oblique and horizontal cell divisions were more frequently observed in mitotic BPs, particularly in the DVR region (where the average angle was 31.7°; Fig. 2C-F,H). A high frequency of oblique or perpendicular cleavage orientations (relative to the ventricular surface) has also been reported in the SVZ of the developing mammalian cortex (Fietz et al., 2010; Garcia-Moreno et al., 2012; Reillo and Borrell, 2012). Live imaging analysis of proliferating cells confirmed the oblique mitotic orientation of the cells on the subapical and basal side of the cultured chicken pallium (Fig. S2). These data suggest that chicken BPs exhibit unique properties during mitosis that resemble some characteristics of mammalian bRGs.

### Manipulation of progenitor dynamics independently increases the number of BPs and Tbr2^+^ cells

The distinct characteristics of BPs and Tbr2^+^ cells in the developing chicken pallium suggest that these cell populations are independent lineages and that their development depends on distinct regulatory mechanisms. Previous studies have shown that LGN (Leu-Gly-Asn repeat-enriched protein; also known as Gpsm2), a regulator of heterotrimeric G proteins, plays an essential role in the determination of cleavage orientation in neural progenitors (Morin et al., 2007; Konno et al., 2008). Experimental manipulation of LGN functions in neural progenitors triggered the delamination of APs, which increased the number of bRG-like cells in the developing mouse cortex (Konno et al., 2008).

**Fig. 3.**
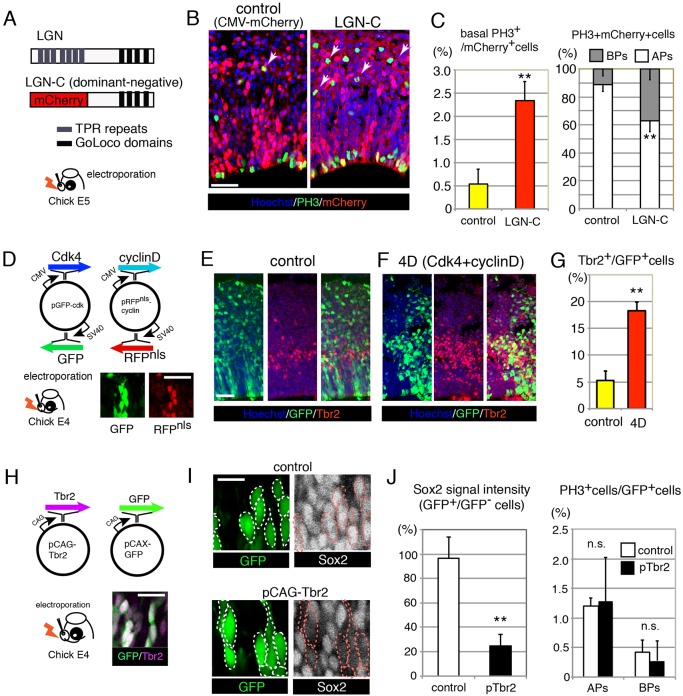
**Experimental amplification of basal mitotic cells and Tbr2^+^ cells in the developing chick pallium.** (A) Diagram of the LGN and LGN-C proteins. C-terminal TPR repeats were replaced with mCherry in LGN-C. (B,C) LGN-C increased the number of PH3^+^ cells on the basal side of the chicken pallium at the expense of APs (arrowheads in B). (D-G) Overexpression of Cdk4 and cyclin D1 (collectively 4D) in the developing chick pallium. (D) The structures of the 4D vectors. Co-expression of GFP and RFP in the same cells was induced by electroporation of 4D vectors. (E-G) Overexpression of 4D vectors increased the number of Tbr2^+^ cells. (H-J) Overexpression of Tbr2 in the developing chicken pallium. (H) Structure of the expression vectors containing GFP and Tbr2. GFP and Tbr2 were co-expressed in the same cells upon electroporation. (I,J) Overexpression of Tbr2 suppressed Sox2 expression in APs but did not change the number of APs and BPs. All bar charts show mean±s.d. **P<0.01 (Student's *t*-test); n.s., not significant. Scale bars: 50 µm in B,E (for E,F); 25 µm in D; 15 µm in H; 10 µm in I.

To address whether knocking down LGN function in the developing chicken pallium promotes the generation of BPs, we introduced an expression vector encoding a dominant-negative form of LGN (LGN-C) into the E5 chick DP (Fig. 3A). Compared with control embryos, into which only an RFP-expressing vector was electroporated, the number of PH3^+^ basal mitotic cells was significantly increased at the expense of apical mitotic cells in the LGN-C-electroporated embryos (Fig. 3B,C, Fig. S3A). These results suggest that the orientation of the mitotic spindle in APs plays a crucial role in the production of basal mitotic cells, which is similar to what occurs in the developing rodent cortex (Konno et al., 2008; Shitamukai et al., 2011; Stahl et al., 2013). However, overexpression of LGN-C did not change the number of Tbr2^+^ cells (data not shown).

In the developing mammalian cortex, expansion of Tbr2^+^ BPs has been induced by overexpression of Cdk4 and cyclin D1 (collectively 4D), which are essential regulators of cell cycle progression in neural progenitors (Lange et al., 2009; Nonaka-Kinoshita et al., 2013). To investigate whether the same experimental manipulations could impact specific cell populations in the developing chicken pallium, we introduced 4D expression vectors into the E5 chick DP (Fig. 3D). The proportion of EdU^+^ cells was significantly increased in 4D-transfected cells compared with untransfected controls (Fig. S3B-F), similar to what has been observed in mouse embryos (Lange et al., 2009). Three days after electroporation, overexpression of 4D vectors dramatically increased the proportion of Tbr2^+^ cells compared with control embryos (Fig. 3E-G). By contrast, the proportion of apical and basal mitotic cells did not differ between the control and 4D-transfected embryos (Fig. S3G,H). This finding is contrary to the effect of overexpressing 4D in the mammalian cortex, which produces an enormous expansion of BPs in the developing mouse and ferret neocortex (Lange et al., 2009; Nonaka-Kinoshita et al., 2013). This discrepancy in phenotypes between mammals and birds might be the result of differences in the characteristics of Tbr2^+^ cells: excess 4D increases the number of Tbr2^+^ cells in both mammals and birds by increasing committed neural precursors (Lange et al., 2009), whereas the proliferative activity of Tbr2^+^ cells is not conserved between these amniote lineages (see also Discussion).

To address the role of Tbr2 in the developing avian pallium, we overexpressed it in the developing chick DP (Fig. 3H). At 24 h after electroporation, a significant reduction was observed in Sox2 expression in the Tbr2-overexpressing cells in the VZ (Fig. 3I,J), although we did not detect an increase of BPs (Fig. 3J), suggesting that Tbr2 promotes neuronal commitment by suppressing Sox2, as is the case in mammalian hippocampal cell lineages (Hodge et al., 2012).

### Non-surface mitosis occurs in other vertebrate pallia

**Fig. 4.**
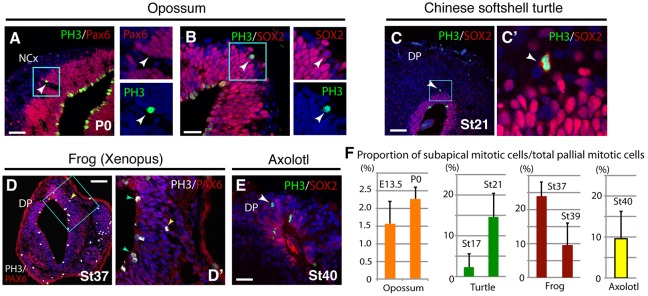
**Comparative analysis of basal mitotic cells in various tetrapod species.** (A,B) Distribution of PH3^+^ cells in the developing opossum cortex (*M. domestica*, P1). PH3^+^ basal mitotic cells (arrowheads) expressed Pax6 (A) or Sox2 (B). (C,C′) A PH3^+^ basal mitotic cell (arrowhead) was Sox2^+^ in the developing turtle cortex. (D,E) Distribution of PH3^+^ cells in developing *Xenopus* (D,D′) and axolotl (E). Arrowheads indicate in green (D′) indicate PH3^+^ cells at the ventricular surface; arrowheads in yellow (D,D′) and white (E) indicate PH3^+^ cells at the basal side of the ventricular zone. (F) The proportion of basal mitotic cells among all pallial mitotic cells in various tetrapods. Mean±range (opposum, turtle, axolotl) or mean±s.d. (*Xenopus*). Scale bars: 100 µm in A,C,D; 50 µm in B,E.

To explore the evolutionary origin of pallial BPs, we examined the distribution of mitotic cells in the developing pallia of various amniote species. Previous studies have shown that a small number of basal mitotic cells exist in the developing marsupial neocortex, although these cells do not express Tbr2 (Cheung et al., 2010; Puzzolo and Mallamaci, 2010). We found that basal mitotic cells in the developing cortex of the opossum (*Monodelphis domestica*) express Pax6 and/or Sox2 (Fig. 4A,B). Basal mitotic cells were also identified in the DP of the developing turtle (*Pelodiscus sinensis*; Fig. 4C), as previously reported in other turtle species (Cheung et al., 2007; Clinton et al., 2015; Martínez-Cerdeño et al., 2015). To extend our analyses to non-amniote vertebrates, we examined the embryonic pallium in amphibians, including the African clawed frog (*Xenopus laevis*) and axolotl (*Ambystoma mexicanum*). Immunostaining with an anti-PH3 antibody revealed that mitotic cells were localized not only at the ventricular surface but also in a subapical position in the embryonic pallia of these amphibian species (Fig. 4D-F). These data suggest that heterogeneity of progenitor subtypes already existed in the common ancestor(s) of amniotes and/or more primitive tetrapods.

## DISCUSSION

### Characteristics of BPs in the chicken pallium

In this study, we revealed the presence of several morphological subtypes of chicken BPs by examining apical and/or basal radial processes during mitosis. Betizeau et al. (2013) have reported that bRGs in the developing primate neocortex include various morphotypes that are classified according to the presence of radial fibers. Importantly, each subtype in the primate neocortex shows bidirectional transitions and all precursor subtypes have self-renewal and neurogenic potentials. Currently, evidence regarding the fates and lineage relationships of chicken BP morphotypes is lacking, including their self-renewal and neurogenic capacities, but these will be clarified in future studies with long-term time-lapse video imaging. bRGs in the developing mammalian neocortex express various transcription factors, including Pax6, Sox2, Tbr2 and Hes1 (Fietz et al., 2010; Hansen et al., 2010; Garcia-Moreno et al., 2012; Reillo and Borrell, 2012; Betizeau et al., 2013). Intriguingly, some of these transcription factors are expressed in mammalian bRGs and chicken BPs, suggesting that a part of the regulatory mechanism that operates in BPs is evolutionary conserved, although Tbr2 expression was rarely detected in chicken BPs.

### Characteristics of Tbr2^+^ cells in the chicken pallium

Here, we show that chicken Tbr2^+^ cells do not display proliferative activity. The number of PH3^+^ or EdU^+^ cells that were also Tbr2^+^ was negligible in the developing chicken pallium (less than 1% and 5%, respectively). Although it is possible that chicken Tbr2^+^ cells exhibit extremely slow cell cycle progression, it is difficult to believe that such a low proliferating potential would have biological relevance given the duration of the neurogenic intervals in the developing chicken pallium. Our data strongly suggest that the majority of Tbr2^+^ cells are not proliferative progenitors but are instead differentiated neurons. This is in stark contrast to the number of Tbr2^+^ intermediate progenitors observed in the developing mouse neocortex [19.3±1.9% of BrdU^+^ cells express Tbr2 at E14.5, and almost all basal PH3^+^ cells express Tbr2 at E14.5 (Englund et al., 2005)]. Interestingly, a previous report indicated that Tbr2^+^ cells in the developing opossum did not show proliferative features, and the authors concluded that these cells represent a postmitotic population in the SVZ (Puzzolo and Mallamaci, 2010). Importantly, the proportion of M-phase and S-phase cells in the Tbr2^+^ population in the chicken pallium is smaller than those in the P10 opossum neocortex (Puzzolo and Mallamaci, 2010). Based on these lines of evidence, we conclude that Tbr2^+^ cells and BPs constitute distinct cell populations in the developing chicken pallium. A recent report also confirmed that Pax6 and Tbr2 do not overlap in the developing chicken pallium, and that Pax6 is expressed in basal mitotic cells (Martinez-Cerdeno et al., 2015).

Previous studies have shown that overexpressing Cdk4 and cyclin D1 (4D) increases Tbr2^+^ BPs in the developing mouse and ferret neocortex (Lange et al., 2009; Nonaka-Kinoshita et al., 2013). Here, we showed that 4D also amplified the number of Tbr2^+^ cells but not the number of BPs in the developing chicken pallium. Thus, the same genetic manipulation triggered distinct phenotypes in the mammalian and avian brain. Considering that the Cdk/cyclin gene families are highly conserved in the animal kingdom, in terms of both sequence and function, it is possible that it is not the molecular functions of 4D but the nature of the neural stem/progenitor cells and/or the cellular compositions in the VZ that differ between mice and chicken. As previously reported, 4D overexpression has stronger effects on Tis21^+^ (also known as Btg2) neurogenic progenitors (Lange et al., 2009); thus, one possibility is that 4D also targets committed precursors and increases their number in the VZ, resulting in the increase of Tbr2^+^ cells in the postmitotic fraction. Currently, we have insufficient evidence about how Tbr2^+^ cells are generated from APs in the chicken VZ. A recent report also revealed a unique manner of neurogenesis in non-mammalian brains involving direct neuronal conversion of neural stem cells (Barbosa et al., 2015), which provides a hint for species-specific behaviors of neural stem/progenitor cells with highly conserved regulatory genes.

### Comparative and evolutionary aspects of BPs in amniotes

Previous reports suggested that pallial BPs evolved independently in mammalian and avian lineages because mammals and birds are distantly related animal groups, and in several groups of (non-avian) reptiles, such as crocodiles and geckos, pallial basal mitotic cells were not detected during embryogenesis (Charvet et al., 2009; Charvet and Striedter, 2011; Nomura et al., 2013). However, recent studies identified abventricular mitotic cells in the developing turtle pallium (Cheung et al., 2007; Martinez-Cerdeno et al., 2015), although this could be a derived feature in the turtle lineage, as is the turtle-specific body plan (Nagashima et al., 2009). Thus, several scenarios are plausible when considering the origin of BPs in amniotes, including: (1) pallial BPs have evolved independently in many amniote groups by *de novo* mechanisms or co-option of subpallial BPs (Charvet et al., 2009); or (2) pallial BPs already existed in the last common ancestors of amniotes but disappeared in some lineages, such as geckos and crocodiles.

**Fig. 5.**
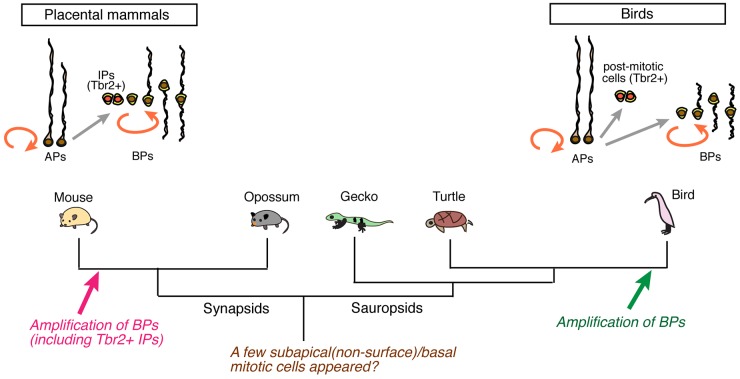
**A possible scenario of changes in pallial progenitor subtypes during amniote evolution.** In mammals, apical progenitors (APs) proliferate and generate basal progenitors (BPs), which include Tbr2^+^ intermediate progenitors (IPs) and other types of BPs [such as basal radial glial cells (bRGs)]. In birds, both BPs and Tbr2^+^ cells exist, but these cells are distinct populations. We propose that a few subapical mitotic cells that have already evolved in ancestral amniotes are at the origin of BPs, although *de novo* BPs appeared in several taxa independently.

Based on the evidence that basal or non-surface mitotic cells exist in a variety of tetrapods, we propose that BPs in placental mammals and birds could be derived from a vestigial population of non-surface mitotic cells that was already present in ancestral amniotes (Fig. 5). Similarly, Tbr2 expression has been reported in various amniotes and amphibians (Brox et al., 2004; Englund et al., 2005; Puzzolo and Mallamaci, 2010; Nomura et al., 2013; Martínez-Cerdeño et al., 2015), suggesting that Tbr2 originally defined committed neuronal lineages in ancestral tetrapods, whereas Tbr2^+^ cells acquired proliferative potential as intermediate progenitors and/or BPs in placental mammalian lineages, which then became a source of neocortical projection neurons (Englund et al., 2005; Sessa et al., 2008; Vasistha et al., 2014). BPs are thought to have played crucial roles in the increase in volume of the cerebral cortex during mammalian evolution (Kriegstein et al., 2006; Martínez-Cerdeño et al., 2006; Borrell and Calegari, 2014; Taverna et al., 2014). In particular, the amplification of bRGs is evident in the outer SVZ of the primate neocortex (Lui et al., 2011; Stahl et al., 2013; Florio et al., 2015). Notably, brain-body scaling has revealed that mammals and birds have relatively larger brains than other vertebrates (Jerison, 1973; Jerison and Barlow, 1985; Striedter, 2005), primarily as a result of the increased volume of the telencephalon in these lineages. Indeed, the expansion of proliferative SVZ compartments in the DVR has been recognized in the enlarged telencephalon of specific avian species (e.g. parrots and zebra finches) (Striedter and Charvet, 2009). Thus, the amplification of BPs commonly contributed to the pallial expansion in various amniotes, although mammals and birds adopted unique strategies for encephalization by increasing the volume of different pallial regions (i.e. the neocortex in mammals and the DVR in birds).

Exploring conserved and derived developmental mechanisms that regulate neural precursors will provide clues to better our understanding of the evolutionary origin of the mammalian neocortex and its homologous structures in other vertebrates.

## MATERIALS AND METHODS

### Animals

Pregnant female mice (CD-1 background) were purchased from Charles River. Fertilized chicken eggs were obtained from a local poultry farm (Yamagishi Farm, Japan) and incubated at 37°C. Pregnant female gray short-tailed opossum (*M. domestica*) were obtained from the breeding colony maintained at Nihon University School of Dentistry, Matsudo, Japan. Colony maintenance and animal experiments on the opossum were approved by Nihon University Animal Care and Use Committee (AP09MD023 and AP12MD015). Fertilized eggs of Chinese soft shell turtles (*P. sinensis*) were obtained from a local breeder (Daiwa Yoshoku, Saga, Japan) and incubated at 28°C. The developmental stages of turtle embryos were determined according to a previous report (Tokita and Kuratani, 2001). Adult *X. laevis* were purchased from a local breeder (Hamamatsu seibutsu kyozai, Shizuoka, Japan) and maintained in a colony at Ehime University. Fertilized eggs of *X. laevis* were incubated at 20°C, and the embryonic stages were determined based on a previous report (Nieuwkoop and Faber, 1967). *A. mexicanum* were purchased from a local breeder and maintained at Ehime University. Experiments on amphibians were approved by Ehime University Animal Care and Use Committee. The fertilized eggs of *A. mexicanum* were placed in fresh water and incubated at 17°C. The embryonic stages of *A. mexicanum* were determined based on a previous report (Schreckenberg and Jacobson, 1975). All experimental procedures of this study were approved by the experimental animal committee of Kyoto Prefectural University of Medicine (M23-272), and were performed in accordance with the relevant guidelines of the committee.

### *In ovo* electroporation

*In ovo* electroporation of the developing chicken pallium was performed according to previous reports (Nomura et al., 2008; Nomura et al., 2013). Briefly, a small window was opened in the shell of incubated eggs, and ∼0.1 µl DNA solution was injected into the lateral ventricle with a small glass needle. Needle-type electrodes (CUY200S, Neppa Gene) were placed on the embryonic head, and square electric pulses (28 V, 50 ms, three times) were applied with a pulse generator (CUY21 EDITII, Neppa Gene). After electroporation, the extraembryonic cavity was filled with sterilized Hank's balanced salt solution (HBSS) containing antibiotics (penicillin/streptomycin and gentamycin), and a window was sealed with Parafilm. Electroporated embryos were incubated at 37°C until analyses. Neural progenitors and neurons were visualized by electroporation of several expression vectors including pCAX-GFP (a gift from Dr Osumi; Inouye et al., 1997), pCAG-mGFP (Addgene, ID14757), pCAGGS-mRFP (a gift from Dr Uchikawa, Osaka University, Osaka, Japan), pmCherry-N1 (Clontech, 632523) and pCALNL-GFP (Addgene, ID13770). Manipulations of gene function were performed with expression vectors including pTK38_mCherry-LGN-C (Addgene, ID46346), pCMS-EGFP-Cdk4, pCMS-EGFP, pDSV-RFPnls-CyclinD, pDSV-RFPnls, pCAG-Eomesodermin-MycDDK (Tbr2; Origene ORF clone MR210179 was subcloned into the *Hin*dIII/*Bgl*I sites of pCAGRB), pCAG-Cre. The expression vectors were dissolved in PBS (2.5-5 µg/µl) mixed with reporter vectors in a 1:1 ratio. To visualize the morphology of mitotic cells, pCAG-Cre was mixed with pCALNL-GFP (0.1 µg/µl and 1 µg/µl, respectively).

### Immunohistochemistry

Brains were fixed in 4% paraformaldehyde (PFA) in phosphate-buffered saline (PBS) at 4°C overnight. After removing the PFA with an excess of PBS, the brains were cryoprotected in a 30% sucrose solution and embedded in Tissue-Tek (Sakura). Frozen sections (14 µm) were sliced on a cryostat (CM1850, Leica) and incubated with primary antibodies: anti-PH3 (mouse monoclonal and rabbit polyclonal; Millipore, 3H10, 05-806, 1:500; 06-570, 1:500), anti-Pax6 (mouse monoclonal; Developmental Studies Hybridoma Bank; 1:10), anti-NeuN (mouse monoclonal; Millipore, MAB377, 1:500), anti-Sox2 (rabbit polyclonal; Abcam, ab97959, 1:500), anti-Tbr2 (rabbit polyclonal; Abcam, ab23345, 1:500), anti-Tbr1 (chicken polyclonal; Millipore, AB2261, 1:500), anti-GFP (rabbit polyclonal; Life Technologies, A11122, 1:500), anti-GFP (rat monoclonal; Nacarai-Tesque, GF090R 04404-84, 1:500) and anti-RFP (rabbit polyclonal; Abcam, ab62341, 1:500). After removal of the primary antibodies by washing in Tris-buffered saline containing 0.01% Tween 20, the sections were incubated with secondary antibodies: Alexa Fluor 488-, 594- and 633-conjugated anti-rabbit, mouse and rat antibodies (Life Technologies; all at 1:500).

Nuclear staining was performed with Hoechst 33258. For amplification of the anti-Tbr2 antibody signal, the sections were incubated with biotinylated anti-rabbit antibody (Vector Laboratories, BA-1000, 1:500), and processed with the ABC Kit (Vector Laboratories) and Tyramide Signal Amplification (TSA) System (PerkinElmer). To examine the morphology of the basal mitotic cells, brains were sectioned with a vibrating microtome (CM3050, Leica). The sections were analyzed using a fluorescence microscope (BX51, Olympus) equipped with a cooled CCD system (DP71, Olympus) and a laser confocal microscope (FV1000D, Olympus).

### Time-lapse imaging of chick embryonic brain slices

An expression vector for GFP (pCAG-EGFP, 1.5 µg/µl) was electroporated into chick embryonic brains *in ovo* at E5. After 24 h, electroporated embryos were dissected in ice-cold Leibovitz's L-15 medium (Sigma), embedded in a 3% low-melting agarose slurry at 35°C and solidified on ice. Agarose blocks containing the telencephalon were soaked in oxygen -saturated HEPES-buffered saline and cut using a microslicer (Linear Slicer Pro7, Dosaka) at a thickness of 300 μm. The slices were transferred onto a membrane filter (Millicell PICMORG50, Millipore) that was placed in a 35 mm glass-based dish (IWAKI, 3910-035). The slice culture medium consisted of Neurobasal medium with B27 supplement (1:50; Invitrogen) and 100 U/ml penicillin/streptomycin. Time-lapse imaging was performed with a confocal scanner box (CV1000, Yokogawa) using a 488 nm excitation and BP525/50 emission filter with a 20× objective lens (LUCPLFLN NA 0.45, Olympus) in 60% O_2_ and 5% CO_2_ at 37°C. The samples were scanned at 512×512 pixels with ten *z*-sections (7 µm intervals) and captured at 10 min intervals for 22 h. The time-lapse data were assembled using CellVoyager CV1000 image analysis software (Yokogawa). In total, five basal mitotic cells were analyzed in two slices.

### Labeling of S-phase

Cells in S-phase were detected using the thymidine analogs BrdU and EdU. Briefly, 0.1 µl BrdU or EdU (10 mg/ml) was injected into the lateral ventricle of developing chick embryos, and the embryos were incubated for 2, 4 or 24 h. For detection of BrdU, brain sections were treated with 2M HCl for 15 min at 37°C and incubated with anti-BrdU antibody (Life Technologies, MoBU-1 B35128, 1:500). EdU was detected with the Click-iT EdU Detection System (Life Technologies).

### Quantifications and statistical analysis

Quantifications of cells was performed under a fluorescence microscope and/or by capturing images with a laser confocal microscope. The images were processed with Photoshop (Adobe) and ImageJ (NIH) software. The signal intensity of Sox2-immunoreactive cells was quantified by measuring the pixel intensities of the cell nuclei and subtracting background intensities. To quantify PH3^+^ mitotic cells among Tbr2^+^ cells (and vice versa), 155 PH3^+^ basal mitotic cells and 1127 Tbr2^+^ cells were examined at E8 (*n*=3), and 620 basal mitotic cells and 2532 Tbr2^+^ cells were examined at E10 (*n*=2). To quantify EdU^+^ cells among Tbr2^+^ cells (and vice versa), 2473 Tbr2^+^ cells and 418 EdU^+^ cells, and 895 Tbr2^+^ cells and 1081 EdU^+^ cells were examined at E8 (*n*=3) and E10 (*n*=2), respectively. In opossum and axolotl, both hemispheres of one sample at each stage were examined, owing to the limited number of samples. At least three animals were examined in each experiment.

For the quantification of mitotic spindle angle data, we confirmed that all groups of data are consistent with a normal (Gaussian) distribution by the Kolmogorov-Smirnov test. Variance of samples was checked by F-test. The statistical significance of mean values was calculated using a parametric Student's *t*-test. Chi-square test was used to compare the proportion of Tbr1^−^ cells among EdU^+^ cells in the SVZ and MZ and the non-neuronal population (Sox2^−^/EdU^+^). For the quantification data in Fig. 3 and Fig. S3, we first checked the homogeneity of variance in each data group by F-test; the statistical significance of mean values was calculated using a parametric Student's *t*-test.

## References

[DEV127100C1] Abdel-Mannan O., Cheung A. F. and Molnár Z. (2008). Evolution of cortical neurogenesis. *Brain Res. Bull.* 75, 398-404. 10.1016/j.brainresbull.2007.10.047 18331905

[DEV127100C2] Arnold S. J., Huang G.-J., Cheung A. F. P., Era T., Nishikawa S.-I., Bikoff E. K., Molnar Z., Robertson E. J. and Groszer M. (2008). The T-box transcription factor Eomes/Tbr2 regulates neurogenesis in the cortical subventricular zone. *Genes Dev.* 22, 2479-2484. 10.1101/gad.475408 18794345 PMC2546697

[DEV127100C3] Barbosa J. S., Sanchez-Gonzalez R., Di Giaimo R., Baumgart E. V., Theis F. J., Gotz M. and Ninkovic J. (2015). Neurodevelopment. Live imaging of adult neural stem cell behavior in the intact and injured zebrafish brain. *Science* 348, 789-793. 25977550 10.1126/science.aaa2729

[DEV127100C4] Betizeau M., Cortay V., Patti D., Pfister S., Gautier E., Bellemin-Ménard A., Afanassieff M., Huissoud C., Douglas R. J., Kennedy H. et al. (2013). Precursor diversity and complexity of lineage relationships in the outer subventricular zone of the primate. *Neuron* 80, 442-457. 10.1016/j.neuron.2013.09.032 24139044

[DEV127100C5] Borrell V. and Calegari F. (2014). Mechanisms of brain evolution: regulation of neural progenitor cell diversity and cell cycle length. *Neurosci. Res.* 86, 14-24. 10.1016/j.neures.2014.04.004 24786671

[DEV127100C6] Borrell V. and Götz M. (2014). Role of radial glial cells in cerebral cortex folding. *Curr. Opin. Neurobiol.* 27, 39-46. 10.1016/j.conb.2014.02.007 24632307

[DEV127100C7] Brox A., Puelles L., Ferreiro B. and Medina L. (2004). Expression of the genes Emx1, Tbr1, and Eomes (Tbr2) in the telencephalon of Xenopus laevis confirms the existence of a ventral pallial division in all tetrapods. *J. Comp. Neurol.* 474, 562-577. 10.1002/cne.20152 15174073

[DEV127100C8] Bulfone A., Martinez S., Marigo V., Campanella M., Basile A., Quaderi N., Gattuso C., Rubenstein J. L. R. and Ballabio A. (1999). Expression pattern of the Tbr2 (Eomesodermin) gene during mouse and chick brain development. *Mech. Dev.* 84, 133-138. 10.1016/S0925-4773(99)00053-2 10473127

[DEV127100C9] Charvet C. J., Owerkowicz T. and Striedter G. F. (2009). Phylogeny of the telencephalic subventricular zone in sauropsids: evidence for the sequential evolution of pallial and subpallial subventricular zones. *Brain Behav. Evol.* 73, 285-294. 10.1159/000230673 19641308

[DEV127100C10] Charvet C. J. and Striedter G. F. (2011). Causes and consequences of expanded subventricular zones. *Eur. J. Neurosci.* 34, 988-993. 21929630 10.1111/j.1460-9568.2011.07818.x

[DEV127100C11] Cheung A. F. P., Pollen A. A., Tavare A., DeProto J. and Molnár Z. (2007). Comparative aspects of cortical neurogenesis in vertebrates. *J. Anat.* 211, 164-176. 10.1111/j.1469-7580.2007.00769.x 17634059 PMC2375772

[DEV127100C12] Cheung A. F. P., Kondo S., Abdel-Mannan O., Chodroff R. A., Sirey T. M., Bluy L. E., Webber N., DeProto J., Karlen S. J., Krubitzer L. et al. (2010). The subventricular zone is the developmental milestone of a 6-layered neocortex: comparisons in metatherian and eutherian mammals. *Cereb. Cortex* 20, 1071-1081. 10.1093/cercor/bhp168 19726493

[DEV127100C13] Cheung A. F., Pollen A. A., Tavare A., DeProto J. and Molnar Z. (2007). Comparative aspects of cortical neurogenesis in vertebrates. *J. Anat.* 211, 164-176. 17634059 10.1111/j.1469-7580.2007.00769.x PMC2375772

[DEV127100C14] Clinton B. K., Cunningham C. L., Kriegstein A. R., Noctor S. C. and Martínez-Cerdeño V. (2015). Radial glia in the proliferative ventricular zone of the embryonic and adult turtle, Trachemys scripta elegans. *Neurogenesis* 1, e970905 10.4161/23262125.2014.970905 PMC4973586 27504470

[DEV127100C15] Dehay C., Kennedy H. and Kosik K. S. (2015). The outer subventricular zone and primate-specific cortical complexification. *Neuron* 85, 683-694. 10.1016/j.neuron.2014.12.060 25695268

[DEV127100C16] Englund C., Fink A., Lau C., Pham D., Daza R. A., Bulfone A., Kowalczyk T. and Hevner R. F. (2005). Pax6, Tbr2, and Tbr1 are expressed sequentially by radial glia, intermediate progenitor cells, and postmitotic neurons in developing neocortex. *J. Neurosci.* 25, 247-251. 10.1523/JNEUROSCI.2899-04.2005 15634788 PMC6725189

[DEV127100C17] Fietz S. A., Kelava I., Vogt J., Wilsch-Bräuninger M., Stenzel D., Fish J. L., Corbeil D., Riehn A., Distler W., Nitsch R. et al. (2010). OSVZ progenitors of human and ferret neocortex are epithelial-like and expand by integrin signaling. *Nat. Neurosci.* 13, 690-699. 10.1038/nn.2553 20436478

[DEV127100C18] Florio M., Albert M., Taverna E., Namba T., Brandl H., Lewitus E., Haffner C., Sykes A., Wong F. K., Peters J. et al. (2015). Human-specific gene ARHGAP11B promotes basal progenitor amplification and neocortex expansion. *Science* 347, 1465-1470. 10.1126/science.aaa1975 25721503

[DEV127100C19] Garcia-Moreno F., Vasistha N. A., Trevia N., Bourne J. A. and Molnar Z. (2012). Compartmentalization of cerebral cortical germinal zones in a lissencephalic primate and gyrencephalic rodent. *Cereb. Cortex* 22, 482-492. 10.1093/cercor/bhr312 22114081

[DEV127100C20] Götz M. and Huttner W. B. (2005). The cell biology of neurogenesis. *Nat. Rev. Mol. Cell Biol.* 6, 777-788. 10.1038/nrm1739 16314867

[DEV127100C21] Hansen D. V., Lui J. H., Parker P. R. L. and Kriegstein A. R. (2010). Neurogenic radial glia in the outer subventricular zone of human neocortex. *Nature* 464, 554-561. 10.1038/nature08845 20154730

[DEV127100C22] Hodge R. D., Nelson B. R., Kahoud R. J., Yang R., Mussar K. E., Reiner S. L. and Hevner R. F. (2012). Tbr2 is essential for hippocampal lineage progression from neural stem cells to intermediate progenitors and neurons. *J. Neurosci.* 32, 6275-6287. 10.1523/JNEUROSCI.0532-12.2012 22553033 PMC3366485

[DEV127100C22a] Inouye S., Ogawa H., Yasuda K., Umesono K. and Tsuji F. (1997). A bacterial cloning vector using a mutated Aequorea green fluorescent protein as an indicator. *Gene* 189, 159-162. 10.1016/S0378-1119(96)00753-6 9168121

[DEV127100C23] Jerison H. J. (1973). *The Evolution of the Brain and Intelligence*. New York: Academic Press.

[DEV127100C24] Jerison H. J. and Barlow H. B. (1985). Animal intelligence as encephalization. *Philos. Trans. R. Soc. B. Biol. Sci.* 308, 21-35. 10.1098/rstb.1985.0007 2858875

[DEV127100C25] Konno D., Shioi G., Shitamukai A., Mori A., Kiyonari H., Miyata T. and Matsuzaki F. (2008). Neuroepithelial progenitors undergo LGN-dependent planar divisions to maintain self-renewability during mammalian neurogenesis. *Nat. Cell Biol.* 10, 93-101. 10.1038/ncb1673 18084280

[DEV127100C26] Kriegstein A., Noctor S. and Martínez-Cerdeño V. (2006). Patterns of neural stem and progenitor cell division may underlie evolutionary cortical expansion. *Nat. Rev. Neurosci.* 7, 883-890. 10.1038/nrn2008 17033683

[DEV127100C27] Lange C., Huttner W. B. and Calegari F. (2009). Cdk4/cyclinD1 overexpression in neural stem cells shortens G1, delays neurogenesis, and promotes the generation and expansion of basal progenitors. *Cell Stem Cell* 5, 320-331. 10.1016/j.stem.2009.05.026 19733543

[DEV127100C28] Lui J. H., Hansen D. V. and Kriegstein A. R. (2011). Development and evolution of the human neocortex. *Cell* 146, 18-36. 10.1016/j.cell.2011.06.030 21729779 PMC3610574

[DEV127100C29] Martínez-Cerdeño V., Noctor S. C. and Kriegstein A. R. (2006). The role of intermediate progenitor cells in the evolutionary expansion of the cerebral cortex. *Cereb. Cortex* 16 Suppl. 1, i152-i161. 10.1093/cercor/bhk017 16766701

[DEV127100C30] Martínez-Cerdeño V., Cunningham C. L., Camacho J., Antczak J. L., Prakash A. N., Cziep M. E., Walker A. I. and Noctor S. C. (2012). Comparative analysis of the subventricular zone in rat, ferret and macaque: evidence for an outer subventricular zone in rodents. *PLoS ONE* 7, e30178 10.1371/journal.pone.0030178 22272298 PMC3260244

[DEV127100C31] Martínez-Cerdeño V., Cunningham C. L., Camacho J., Keiter J. A., Ariza J., Lovern M. and Noctor S. C. (2015). Evolutionary origin of Tbr2-expressing precursor cells and the subventricular zone in the developing cortex. *J. Comp. Neurol*. 10.1002/cne.23879 PMC4843790 26267763

[DEV127100C32] Morin X., Jaouen F. and Durbec P. (2007). Control of planar divisions by the G-protein regulator LGN maintains progenitors in the chick neuroepithelium. *Nat. Neurosci.* 10, 1440-1448. 10.1038/nn1984 17934458

[DEV127100C33] Nagashima H., Sugahara F., Takechi M., Ericsson R., Kawashima-Ohya Y., Narita Y. and Kuratani S. (2009). Evolution of the turtle body plan by the folding and creation of new muscle connections. *Science* 325, 193-196. 19590000 10.1126/science.1173826

[DEV127100C34] Nieuwkoop P. D. and Faber J. (1967). *Normal Table of Xenopus laevis (Daudin). A Systematical and Chronological Survey of the Development from the Fertilized Egg till the End of Metamorphosis*. Amsterdam: North-Holland Publishing Company.

[DEV127100C35] Nomura T., Takahashi M., Hara Y. and Osumi N. (2008). Patterns of neurogenesis and amplitude of Reelin expression are essential for making a mammalian-type cortex. *PLoS ONE* 3, e1454 10.1371/journal.pone.0001454 18197264 PMC2175532

[DEV127100C36] Nomura T., Gotoh H. and Ono K. (2013). Changes in the regulation of cortical neurogenesis contribute to encephalization during amniote brain evolution. *Nat. Commun.* 4, 2206 10.1038/ncomms3206 23884180

[DEV127100C38] Nonaka-Kinoshita M., Reillo I., Artegiani B., Ángeles Martínez-Martínez M., Nelson M., Borrell V. and Calegari F. (2013). Regulation of cerebral cortex size and folding by expansion of basal progenitors. *EMBO J.* 32, 1817-1828. 10.1038/emboj.2013.96 23624932 PMC3926188

[DEV127100C39] Pilz G.-A., Shitamukai A., Reillo I., Pacary E., Schwausch J., Stahl R., Ninkovic J., Snippert H. J., Clevers H., Godinho L. et al. (2013). Amplification of progenitors in the mammalian telencephalon includes a new radial glial cell type. *Nat. Commun.* 4, 2125 10.1038/ncomms3125 23839311 PMC3717501

[DEV127100C40] Puelles L. (2001). Thoughts on the development, structure and evolution of the mammalian and avian telencephalic pallium. *Philos. Trans. R. Soc. B Biol. Sci.* 356, 1583-1598. 10.1098/rstb.2001.0973 PMC1088538 11604125

[DEV127100C41] Puzzolo E. and Mallamaci A. (2010). Cortico-cerebral histogenesis in the opossum Monodelphis domestica: generation of a hexalaminar neocortex in the absence of a basal proliferative compartment. *Neural Dev.* 5, 8 10.1186/1749-8104-5-8 20302607 PMC2859365

[DEV127100C42] Reillo I. and Borrell V. (2012). Germinal zones in the developing cerebral cortex of ferret: ontogeny, cell cycle kinetics, and diversity of progenitors. *Cereb. Cortex* 22, 2039-2054. 10.1093/cercor/bhr284 21988826

[DEV127100C43] Reillo I., de Juan Romero C., Garcia-Cabezas M. A. and Borrell V. (2011). A role for intermediate radial glia in the tangential expansion of the mammalian cerebral cortex. *Cereb. Cortex* 21, 1674-1694. 10.1093/cercor/bhq238 21127018

[DEV127100C44] Schreckenberg G. M. and Jacobson A. G. (1975). Normal stages of development of the axolotl Ambystoma mexicanum. *Dev. Biol.* 42, 391-400. 1167837 10.1016/0012-1606(75)90343-7

[DEV127100C45] Sessa A., Mao C.-a., Hadjantonakis A. K., Klein W. H. and Broccoli V. (2008). Tbr2 directs conversion of radial glia into basal precursors and guides neuronal amplification by indirect neurogenesis in the developing neocortex. *Neuron* 60, 56-69. 10.1016/j.neuron.2008.09.028 18940588 PMC2887762

[DEV127100C46] Shitamukai A., Konno D. and Matsuzaki F. (2011). Oblique radial glial divisions in the developing mouse neocortex induce self-renewing progenitors outside the germinal zone that resemble primate outer subventricular zone progenitors. *J. Neurosci.* 31, 3683-3695. 10.1523/JNEUROSCI.4773-10.2011 21389223 PMC6622781

[DEV127100C47] Stahl R., Walcher T., De Juan Romero C., Pilz G. A., Cappello S., Irmler M., Sanz-Aquela J. M., Beckers J., Blum R., Borrell V. et al. (2013). Trnp1 regulates expansion and folding of the mammalian cerebral cortex by control of radial glial fate. *Cell* 153, 535-549. 10.1016/j.cell.2013.03.027 23622239

[DEV127100C48] Striedter G. F. (2005). *Principle of Brain Evolution*. Sunderland: Sinauer Associates.

[DEV127100C49] Striedter G. F. and Charvet C. J. (2009). Telencephalon enlargement by the convergent evolution of expanded subventricular zones. *Biol. Lett.* 5, 134-137. 10.1098/rsbl.2008.0439 18842571 PMC2657736

[DEV127100C50] Suzuki I. K., Kawasaki T., Gojobori T. and Hirata T. (2012). The temporal sequence of the mammalian neocortical neurogenetic program drives mediolateral pattern in the chick pallium. *Dev. Cell* 22, 863-870. 10.1016/j.devcel.2012.01.004 22424929

[DEV127100C51] Taverna E., Götz M. and Huttner W. B. (2014). The cell biology of neurogenesis: toward an understanding of the development and evolution of the neocortex. *Annu. Rev. Cell Dev. Biol.* 30, 465-502. 10.1146/annurev-cellbio-101011-155801 25000993

[DEV127100C52] Tokita M. and Kuratani S. (2001). Normal embryonic stages of the chinese softshelled turtle Pelodiscus sinensis (Trionychidae). *Zool. Sci.* 18, 705-715.

[DEV127100C53] Vasistha N. A., Garcia-Moreno F., Arora S., Cheung A. F., Arnold S. J., Robertson E. J. and Molnar Z. (2014). Cortical and clonal contribution of Tbr2 expressing progenitors in the developing mouse brain. *Cereb. Cortex* 25, 3290-3302. 24927931 10.1093/cercor/bhu125 PMC4585488

[DEV127100C54] Wang X., Tsai J.-W., LaMonica B. and Kriegstein A. R. (2011). A new subtype of progenitor cell in the mouse embryonic neocortex. *Nat. Neurosci.* 14, 555-561. 10.1038/nn.2807 21478886 PMC3083489

